# Effects of insulin degludec and insulin glargine on day-to-day fasting plasma glucose variability in individuals with type 1 diabetes: a multicentre, randomised, crossover study

**DOI:** 10.1007/s00125-015-3648-y

**Published:** 2015-06-05

**Authors:** Tomoaki Nakamura, Kazuhiko Sakaguchi, Anna So, Shinsuke Nakajima, Michinori Takabe, Hisako Komada, Yoko Okuno, Yushi Hirota, Takehiro Nakamura, Keiji Iida, Michiko Kajikawa, Masao Nagata, Wataru Ogawa, Susumu Seino

**Affiliations:** Division of Diabetes and Endocrinology, Department of Internal Medicine, Kobe University Graduate School of Medicine, 7-5-1 Kusunoki-cho, Chuo-ku, Kobe, 650-0017 Japan; Department of Internal Medicine, Shinsuma General Hospital, Kobe, Japan; Department of Internal Medicine, Kobe City Hospital Organization, Kobe City Medical Center West Hospital, Kobe, Japan; Department of Internal Medicine, Hyogo Prefectural Kakogawa Medical Center, Kakogawa, Japan; Department of Internal Medicine, Yodogawa Christian Hospital, Osaka, Japan; Department of Internal Medicine, Kakogawa City Hospital Organization, Kakogawa City West Hospital, Kakogawa, Japan; Department of Internal Medicine, Division of Molecular and Metabolic Medicine, Department of Physiology and Cell Biology, Kobe University Graduate School of Medicine, Kobe, Japan

**Keywords:** Basal-bolus insulin therapy, Day-to-day fasting plasma glucose variability, Insulin degludec, Insulin glargine, Type 1 diabetes

## Abstract

**Aims/hypothesis:**

We compared the effects of insulin degludec (IDeg; Des(B30)LysB29(γ-Glu Nε-hexadecandioyl) human insulin) and insulin glargine (IGlar; A21Gly,B31Arg,B32Arg human insulin) on the day-to-day variability of fasting plasma glucose (FPG) levels in individuals with type 1 diabetes treated with basal-bolus insulin injections.

**Methods:**

The effects of basal-bolus insulin therapy for 4 weeks with either IDeg or IGlar as the basal insulin in adult C-peptide-negative outpatients with type 1 diabetes were investigated in an open-label, multicentre, randomised, crossover trial. Randomisation was conducted using a centralised allocation process. The primary endpoints were the SD and CV of FPG during the final week of each treatment period. Secondary endpoints included serum glycoalbumin level, daily dose of insulin, intraday glycaemic variability and frequency of severe hypoglycaemia.

**Results:**

Thirty-six randomised participants (17 in the IDeg/IGlar and 19 in the IGlar/IDeg groups) were recruited, and data for 32 participants who completed the trial were analysed. The mean (7.74 ± 1.76 vs 8.56 ± 2.06 mmol/l; *p* = 0.04) and SD (2.60 ± 0.97 vs 3.19 ± 1.36 mmol/l; *p* = 0.03) of FPG were lower during IDeg treatment than during IGlar treatment, whereas the CV did not differ between the two treatments. The dose of IDeg was smaller than that of IGlar (11.0 ± 5.2 vs 11.8 ± 5.6 U/day; *p* < 0.01), but other secondary endpoints did not differ between the treatments.

**Conclusions/interpretation:**

IDeg yielded a lower FPG level and smaller day-to-day variability of FPG at a lower daily dose compared with IGlar in participants with type 1 diabetes. IDeg serves as a good option for basal insulin in the treatment of type 1 diabetes.

*Trial registration*: University Hospital Medical Information Network 000009965.

*Funding*: This research recieved no specific grant from any funding agency in the public, commercial or not-for-profit sectors.

## Introduction

The basal secretion of insulin during the fasting state plays an essential role in maintaining an appropriate level of endogenous glucose production, and the additional secretion of the hormone after a meal is critical for the anabolism and storage of this energy source. Individuals with type 1 diabetes, in whom insulin secretion is greatly impaired, must therefore supplement their endogenous insulin by a basal-bolus administration of exogenous hormone in order to mimic the physiological regulation of energy metabolism and improve glycaemic control.

In patients with type 1 diabetes who undergo basal-bolus insulin therapy with multiple daily injections, basal insulin is largely responsible for the stability of blood glucose levels in the fasting state. Insulin glargine (IGlar; A21Gly,B31Arg,B32Arg human insulin) is a long-acting insulin analogue that is widely administered as a basal insulin in basal-bolus therapy. IGlar exhibits a longer and flatter temporal pattern of hormone action than NPH insulin, with a duration of biological action of ∼24 h [1]. Evidence suggest that basal-bolus therapy for patients with type 1 diabetes using IGlar has been associated with a reduced number of daily injections [2, 3], a reduced frequency of hypoglycaemia [3–7], a reduced variability of fasting plasma glucose (FPG) concentration [2] and lower FPG and HbA_1c_ levels [4, 6–9] relative to NPH insulin, which is indicative of the benefits of IGlar for such therapy.

Insulin degludec (IDeg; Des(B30)LysB29(γ-Glu Nε-hexadecandioyl) human insulin) is a novel ultra-long-acting insulin analogue that was first launched in the UK in January 2013 and is now available in several European and Asian countries including Japan. The duration of action for IDeg, estimated at ∼42 h [10], is much longer than that for IGlar. Such pharmacokinetics are attributable both to the slower absorption of IDeg from the injection site into the circulation as a result of its formation of soluble multihexameric chains and to its prolonged retention in the circulation as a result of its binding to albumin in the blood [11]. Clinical studies have revealed that basal-bolus therapy with IDeg is associated with a similar level of glycaemic control [12, 13], a lower daily dose [13, 14] and less frequent nocturnal hypoglycaemia [12–14] in individuals with type 1 diabetes in comparison with IGlar. The frequency of nocturnal hypoglycaemia has been shown to be related to the variability of FPG levels [15]. Moreover, a euglycaemic glucose clamp analysis has revealed a lower variability in pharmacodynamics for IDeg than for IGlar [16]. Treatment with IDeg might thus be expected to result in lower day-to-day variability in the glucose-lowering effect of basal-bolus therapy. There has, however, been no clinical study to date that has directly evaluated this possibility.

We performed the current study to investigate the day-to-day variability of the glucose-lowering effect of IDeg. We compared the SD and CV of FPG levels in individuals with type 1 diabetes treated with basal-bolus insulin therapy using either IDeg or IGlar.

## Methods

This study was conducted in accordance with the Declaration of Helsinki and was approved by the local institutional review boards of the participating centres. Written informed consent was obtained from all participants before beginning the trial, which has been registered with the University Hospital Medical Information Network (UMIN 000009965). The study was an open-label, randomised, multicentre, crossover trial designed to investigate the day-to-day variability of FPG in participants with type 1 diabetes treated with basal-bolus insulin therapy using either IDeg or IGlar as the basal insulin. The participating centres and principal investigators for the trial are listed in the Appendix.

Inclusion criteria for the trial included: (1) individuals with type 1 diabetes who were at least 18 years of age and whose serum C-peptide concentration had been confirmed to be below detectable levels (<0.07 nmol/l) at least twice; (2) individuals who had been treated for at least 1 year with basal-bolus insulin injections with IGlar as the basal insulin and a rapid-acting insulin analogue or regular insulin as the bolus insulin; (3) individuals with the ability to perform self-monitoring of blood glucose (SMBG); and (4) individuals with an HbA_1c_ level of ≤8% (64 mmol/mol). The exclusion criteria included: (2) the use of medications that affect glucose metabolism (such as beta-blockers, corticosteroids and monoamine oxidase inhibitors); (2) a history of myocardial infarction, angina pectoris, coronary artery bypass surgery or heart failure within the preceding 6 months; (3) severe hypertension (systolic BP of ≥180 mmHg or diastolic BP of ≥100 mmHg); (4) severe liver dysfunction (serum aspartate aminotransferase or alanine aminotransferase levels of ≥2.5 times the normal upper limit); (5) severe renal dysfunction (a serum creatinine concentration of ≥177 μmol/l); (6) the presence of antibodies to insulin that might influence the variability of plasma glucose levels; (7) frequently recurring severe hypoglycaemia or hospitalisation due to serious hypoglycaemia or diabetic ketoacidosis within the previous year; (8) the complication of proliferative diabetic retinopathy with a high risk of haemorrhage; (9) existing or possible pregnancy or breastfeeding; (10) the presence of cancer; (11) a complicating psychiatric disorder; (12) alcoholism or other drug addiction; and (13) an investigator’s declaration that the participant was otherwise inappropriate for the study. Insulin antibodies were checked for all participants as part of the screening procedure. Severe hypoglycaemia was defined as events associated with central nervous system manifestations during which the patient required the assistance of another person. The judgement of frequent recurrence of hypoglycaemia was made by each attending physician.

**Fig. 1:**
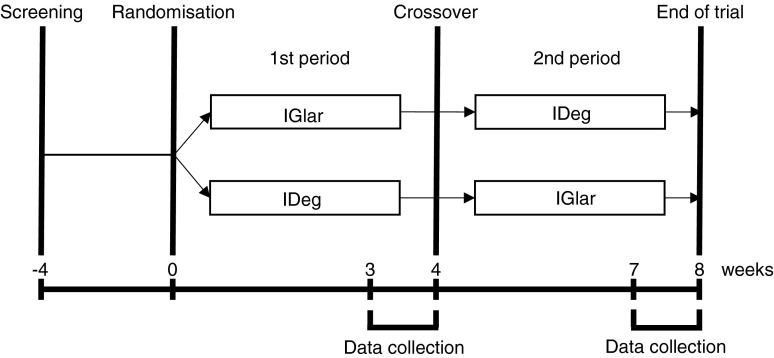
Study design. Eligible patients were randomly allocated to the IGlar/IDeg group (upper arm) or the IDeg/IGlar group (lower arm). In the IGlar/IDeg group, the basal insulin was switched after 4 weeks from IGlar to IDeg, whereas in the IDeg/IGlar group the basal insulin was switched after 4 weeks from IDeg to IGlar. The last week of each treatment period constituted the data collection phase in which seven SMBG measurements per day were performed and the insulin dosage was determined. The serum glycoalbumin level was also measured on the last day of each treatment period

Individuals found to satisfy the criteria were randomly assigned to the IGlar (first period)/IDeg (second period) (IGlar/IDeg) or the IDeg (first period)/IGlar (second period) (IDeg/IGlar) group by a centralised allocation process. In the IGlar/IDeg group, the basal insulin was switched after 4 weeks from IGlar (Lantus, SoloSTAR; Sanofi, Paris, France) to IDeg (Tresiba, FlexTouch; Novo Nordisk, Bagsvaerd, Denmark). In the IDeg/IGlar group, the basal insulin was switched after 4 weeks from IDeg to IGlar. The participants were directed to determine their plasma glucose level four times a day (before breakfast, lunch and dinner as well as at bedtime) by SMBG during the entire trial period. The last week of each treatment period constituted the data collection phase, during which the participants were directed to determine their plasma glucose level seven times a day (before breakfast, 2 h after breakfast, before lunch, 2 h after lunch, before dinner, 2 h after dinner and at bedtime) (Fig. 1). All individuals were provided with the same device (OneTouch Ultra; Johnson & Johnson, New Brunswick, NJ) and directed to perform blood testing with it. Given that the measurements of the SMBG device are calibrated to the plasma glucose concentrations, we considered the values recorded by this device as the plasma glucose levels. The serum glycoalbumin level was measured at the end of each treatment period.

Basal insulin was administered at the same time of day in both treatment periods. The initial dose of IDeg was equivalent to 80% of the IGlar dose that had been administered before the start of the trial. Insulin titration was then performed according to the attending physician’s instruction or the patient’s own judgement to achieve the target plasma glucose level. The type of bolus insulin preparation was not changed during the trial, and the preprandial bolus insulin dose was adjusted by each participant. The target plasma glucose level before breakfast, lunch and dinner as well as at bedtime was initially <7.21 mmol/l and was subsequently reduced to <6.11 mmol/l for individuals capable of achieving a reduction. The participants were directed to avoid hypoglycaemia (<3.89 mmol/l) at any time during the day.

The primary endpoint of the study was the day-to-day variability of FPG level as evaluated by the SD and CV of the plasma glucose level determined by SMBG before breakfast during the last week of each 4 week treatment period (Fig. 1). Secondary endpoints included the serum glycoalbumin level on the final day of each treatment period, the administered insulin dose (the mean for the last week of each treatment period), the intraday glycaemic variability calculated from the seven daily measurements of plasma glucose during the final week of each treatment period, and the frequency of severe hypoglycaemic events (defined as events associated with central nervous system manifestations during which the patient required the assistance of another person).

To determine the sample size, we performed a preliminary analysis of the day-to-day variability of FPG in individuals with type 1 diabetes treated with basal-bolus insulin therapy including IGlar as the basal insulin at Kobe University Hospital. The mean CV and mean SD of the CV of FPG for 34 participants over 7 days were 36.1% and 16.5%, respectively. The number of individuals for whom a 30% alteration of the mean CV could be detected with an *α* value of 0.05 and with a statistical power of 80% was 32. We assumed that 10% of those recruited might drop out from the trial and therefore determined the total number of participants required to be 36.

We evaluated normal distributions of the data with the use of Shapiro–Wilk test, and non-normally distributed data were transformed to satisfy the normality assumption by Box–Cox transformation. Data are presented as means ± SD and were analysed with repeated-measures ANOVA and Grizzle’s model for a 2 × 2 crossover study. In these statistical methods, the values are analysed in a paired manner, using each patient as their own control. The statistical analyses were performed using the SPSS version 22 software package (IBM, Armonk, NY, USA). A *p* value <0.05 was considered statistically significant.

## Results

The total of 36 randomised participants consisted of 19 individuals in the IGlar/IDeg group and 17 individuals in the IDeg/IGlar group. Three participants in the IDeg/IGlar group did not want to change their insulin preparation at the time of crossover, and they withdrew consent for participation in the study at this time. One participant in the IGlar/IDeg group who initially agreed to participate in the study during the screening period withdrew consent at the beginning of the first period of allocated insulin administration. These four participants were excluded, so a total of 32 participants who completed the trial were therefore included in the analysis. The clinical characteristics of these 32 participants are shown in Table 1.

**Table 1:** Clinical characteristics of the participants according to study group

Variable	
Number of participants	32
Age (years)	57 ± 14
Male/female	13/19
Duration of diabetes (years)	18 ± 10
BMI (kg/m^2^)	22.6 ± 3.2
HbA_1c_	
(%)	7.4 ± 0.8
(mmol/mol)	54.7 ± 9.2
Serum glycoalbumin (%)	22.1 ± 3.7
BP (mmHg)	
Systolic	126 ± 11
Diastolic	70 ± 9
Complications (%)	
Retinopathy	37.5
Neuropathy	34.5
Nephropathy	31.3

Data for continuous variables are means ± SD

At the start of the trial, the mean total daily dose of insulin was 32.7 ± 12.4 U/day, the mean daily dose of basal insulin was 12.1 ± 5.9 U/day, and the mean daily dose of bolus insulin was 20.9 ± 8.8 U/day. The number of participants who administered IGlar in the morning, at noon, in the evening and at bedtime was 9, 6, 3 and 14, respectively.

We first examined interaction effects (periods and carry-over effects) for the mean, SD and CV of FPG with repeated-measures ANOVA and Grizzle’s model for a 2 × 2 crossover study, and found no interaction effects between the two intervention periods (*p* = 0.22, 0.44 and 0.80, respectively).

**Fig. 2:**
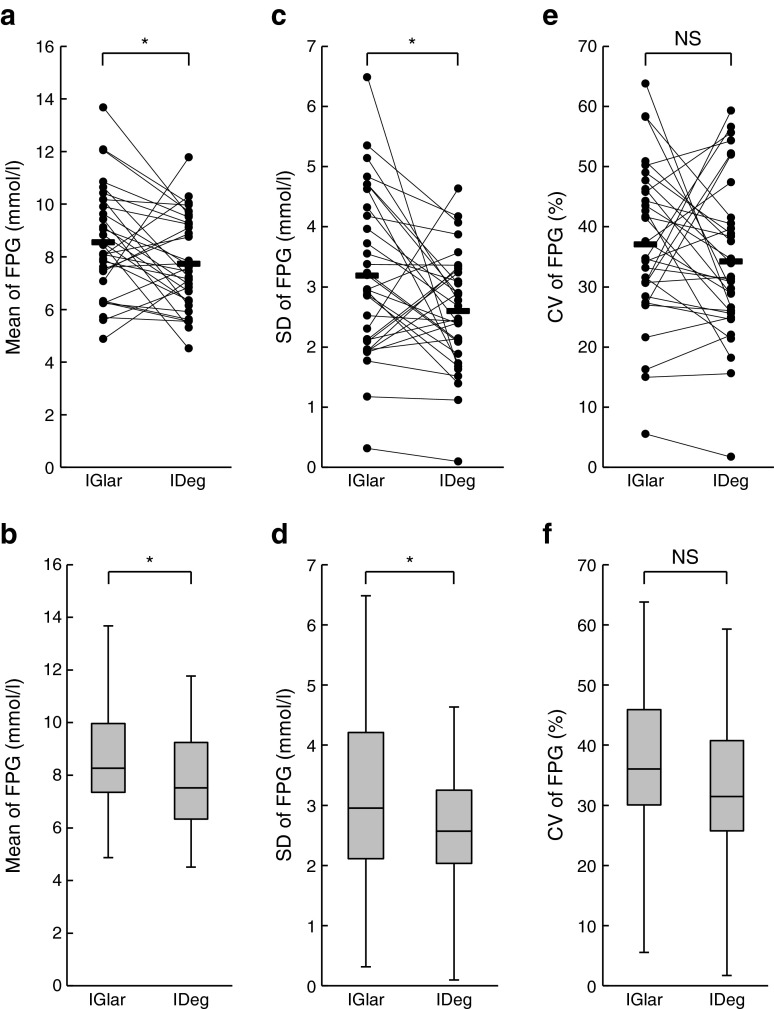
Mean and variability of FPG levels. The mean (**a**), SD (**c**) and CV (**e**) of FPG levels during the IGlar and IDeg treatment periods were determined for each participant. The two data points for a particular individual were connected by a line. Horizontal bars represent the corresponding mean values for all participants. Box plots of the mean (**b**), SD (**d**) and CV (**f**) of FPG are shown. The line within each box represents the median, and the top and bottom of the box represent the 75th and 25th percentiles, respectively. The whiskers indicate the maximum and minimum values. *p < 0.05. NS, not significant

The mean FPG level determined before breakfast during the data collection phase was significantly lower in the IDeg administration period than in the IGlar administration period (7.74 ± 1.76 vs 8.56 ± 2.06 mmol/l; *p* = 0.04) (Fig. 2a, b). The SD of FPG was also significantly smaller during IDeg administration than during IGlar administration (2.60 ± 0.97 vs 3.19 ± 1.36 mmol/l; *p* = 0.03) (Fig. 2c, d). The CV of FPG did not, however, differ between the two treatment periods (34.3 ± 13.3 vs 37.1 ± 13.0% for IDeg and IGlar, respectively; *p* = 0.32) (Fig. 2e, f).

The mean of the seven daily plasma glucose measurements was significantly lower during the IDeg administration period than the IGlar administration period (8.30 ± 1.50 vs 9.13 ± 1.92 mmol/l; *p* < 0.01). Among the seven measurement points, a significant difference between the two treatment periods was apparent only for that before breakfast. The intraday SD (3.39 ± 0.94 vs 3.13 ± 0.85 mmol/l for IGlar and IDeg) and intraday CV (38.2 ± 9.2 vs 37.6 ± 8.1% for IGlar and IDeg) for the seven measurements were similar in the two treatment periods (*p* = 0.16 and 0.64, respectively).

To further analyse the plasma glucose variability during the two treatment periods, we calculated the SD of all the plasma glucose values from all the days of the last week of treatment (SD_*T*_), the SD of the daily means of plasma glucose (SD_*dm*_) and the SD between days within specified time points averaged over all times of day (SD_*b*_) (Table 2), as have been previously used to evaluate glucose variability [17]. We also calculated the mean difference in plasma glucose values before and after breakfast [mean(|Δ_ab–bb_|)], lunch [mean(|Δ_al–bl_|)] and dinner [mean(|Δ_ad–bd_|)], the mean difference in plasma glucose values before and after all meals [(mean(|Δ_overall average_|)] and the mean difference between the bedtime plasma glucose and FPG [mean(|Δ_bb–b_|)] (Table 2). Of these variables, only SD_*T*_ was significantly smaller for IDeg than for IGlar (3.85 ± 0.91 vs 3.47 ± 0.86 mmol/l for IGlar and IDeg, respectively; *p* = 0.04). The serum glycoalbumin level was also similar for the two treatment periods (21.8 ± 3.6 vs 21.5 ± 3.0% for IGlar and IDeg, respectively; *p* = 0.40). No episodes of severe hypoglycaemia occurred during the entire trial period.

**Table 2:** Variables for glycaemic variability

	*n*	IGlar	IDeg	*p* values
SD_*T*_ (mmol/l)	32	3.85 ± 0.91	3.47 ± 0.86	0.04
SD_*dm*_ (mmol/l)	32	2.04 ± 1.07	1.78 ± 0.65	0.25
SD_*b*_ (mmol/l)	32	3.29 ± 1.05	3.00 ± 0.80	0.10
Mean(|Δ_ab–bb_|) (mmol/l)	17	3.42 ± 1.38	3.84 ± 1.94	0.34
Mean(|Δ_al–bl_|) (mmol/l)	22	4.20 ± 2.78	3.62 ± 1.54	0.44
Mean(|Δ_ad–bd_|) (mmol/l)	21	4.54 ± 2.66	3.46 ± 1.27	0.11
Mean(|Δ_overall average_|) (mmol/l)	23	4.25 ± 2.08	3.87 ± 1.17	0.49
Mean(|Δ_bb–b_|) (mmol/l)	28	4.08 ± 1.43	4.24 ± 1.84	0.76

Data are means ± SD

The total daily dose of insulin was slightly lower in the IDeg administration period than in the IGlar administration period, although this difference was not statistically significant (31.2 ± 11.4 vs 31.9 ± 11.9 U/day; *p* = 0.15). The daily dose of basal insulin in the IDeg period was slightly but significantly lower than that in the IGlar period (11.0 ± 5.2 vs 11.8 ± 5.6 U/day; *p* < 0.01). The daily dose of bolus insulin was similar in the two treatment periods (20.5 ± 8.5 vs 20.5 ± 8.6 U/day for IGlar and IDeg, respectively; *p* = 0.68). During the study period, severe hypoglycaemia or other notable adverse effects including skin rash, liver or kidney dysfunctions, abnormalities in electrolytes or in completely blood count were not reported.

## Discussion

The primary endpoints of this study were the SD and CV of FPG during treatment with IDeg and IGlar in a randomised crossover trial in individuals with type 1 diabetes who underwent basal-bolus insulin therapy. We have shown here that the SD of FPG levels was smaller during treatment with IDeg than with IGlar. The CV of FPG did not, however, differ between the two treatments, probably as a result of the smaller mean value for the IDeg treatment period. Given that the FPG concentration is greatly influenced by the action of basal insulin, our results indicate that day-to-day variability in the glucose-lowering effect of IDeg is smaller than that for IGlar. Clinical trials that have compared the efficacy of IDeg with that of IGlar have revealed a lower frequency of nocturnal hypoglycaemia during IDeg treatment, whereas the frequency of hypoglycaemia during the daytime was similar during treatment with IDeg and IGlar [13, 14]. The variability in FPG levels is closely related to the frequency of nocturnal hypoglycaemia in patients with type 1 and type 2 diabetes [15]. It is thus possible that the reduced frequency of nocturnal hypoglycaemia observed during IDeg treatment in previous studies is attributable to the low variability of the glucose-lowering effect of this insulin analogue revealed by the present study.

Although we set the same target plasma glucose level during both treatment periods, the FPG concentration was lower during IDeg treatment than IGlar treatment, whereas the daily dose of basal insulin was smaller during IDeg treatment. We cannot completely exclude the possibility that these observations are attributable to incidental bias. However, lower FPG levels achieved with a lower daily dose of basal insulin in spite of the same plasma glucose target were also apparent during treatment with IDeg in comparison with IGlar in a previous study [13]. The pharmacological characteristics of IDeg may thus contribute to these observations. In the present study, the participants themselves titrated the dose of basal insulin in order to achieve the target FPG level. If the variability of the glucose-lowering effect is small, it is easier to titrate the dose of basal insulin in order to maintain the FPG close to the target level. Moreover, the temporal pattern of the biological action of IDeg has been found to be flatter than that of IGlar [16], which might also be related to the reduced frequency of nocturnal hypoglycaemia that was observed during treatment with IDeg [13, 14]. The ‘peak-less’ characteristic of the pharmacokinetics of IDeg might thus allow patients to achieve the target FPG level with a smaller dosage. In this regard, the proportion of participants who achieved the primary target plasma glucose level (<7.21 mmol/l) tended to be higher during IDeg treatment than IGlar treatment (44% and 25%, respectively), although this difference was not statistically significant (*p* = 0.19). In this study, the total basal dose was approximately 35% (35.9% and 34.6% for IGlar and IDeg, respectively) of the total daily dose at the end of each treatment period. This percentage was lower than that previously reported for white individuals with type 1 diabetes, but was similar to those reported for Japanese patients with type 1 diabetes [18, 19].

In the present study, we recruited only participants with type 1 diabetes whose serum C-peptide level was below the limit of detection, given that the pharmacology of basal insulin is well reflected by the plasma glucose level in such individuals. Moreover, all the participants in our study were non-obese Japanese adults. Whether the results of the present study are applicable to patients whose capacity for insulin secretion is not completely exhausted or to those with different physical constitutions or ethnicities thus remains to be determined.

The current study has some limitations. First, this trial was an open-label study, and we could not exclude possible biases induced by recognition of the insulin preparations. Moreover, the injection devices were different for each of the insulin preparations. Given that all the participants had used IGlar before the study, they were familiar with IGlar and had to familiarise themselves with the IDeg. We cannot exclude the possibility that this may have introduced some bias into the study. Finally, the proportions of patients who achieved the target FPG levels were low in both treatment periods.

In summary, our results indicate that the day-to-day variability in glucose-lowering effect is smaller for IDeg than for IGlar. The current result might be related to a lower frequency of nocturnal hypoglycaemia in individuals with type 1 diabetes who use IDeg as basal insulin [12–14]. It remains to be elucidated whether the insulin preparation has beneficial effects on long-term glycaemic control and on diabetic complications.

## Appendix

Principal investigators at the participating centres are as follows: Kazuhiko Sakaguchi (Kobe University Hospital, Obara Hospital), Takeshi Ohara (Hyogo Prefectural Kakogawa Medical Center), Tomoaki Nakamura (Akashi Medical Center), Michinori Takabe (Shinsuma General Hospital), Takehiro Nakamura (Kobe City Hospital Organization, Kobe City Medical Center West Hospital), Michiko Kajikawa (Yodogawa Christian Hospital), and Masao Nagata (Kakogawa City Hospital Organization, Kakogawa City West Hospital).

## Abbreviations

FPG: Fasting plasma glucose
IDeg: Insulin degludec
IGlar: Insulin glargine
SMBG: Self-monitoring of blood glucose
